# Integrated Experimental and Computational Analysis of SLM-Fabricated Ti6Al4V Octet-Truss Scaffolds for Bone Tissue Engineering

**DOI:** 10.3390/ma19081646

**Published:** 2026-04-20

**Authors:** Dmitriy Dogadkin, Bagdat Azamatov, Suresh Alapati, Daniyar Kaliyev, Sergey Rudenko, Marzhan Sadenova, Nikolay Dmitriev

**Affiliations:** 1 «Smart Engineering» Competence Center, D. Serikbayev East Kazakhstan Technical University, D. Serikbayev St. 19, Ust-Kamenogorsk 070004, Kazakhstan; ddogadkin@edu.ektu.kz (D.D.); bazamatov@edu.ektu.kz (B.A.); dkaliyev@ektu.kz (D.K.); srudenko@edu.ektu.kz (S.R.); 2 «ALTAI East Kazakhstan Regional Technopark» LLP, D. Serikbayev St. 37, Ust-Kamenogorsk 070010, Kazakhstan; 3 Department of Mechatronics Engineering, Kyungsung University, 309, Suyeong-ro (Daeyeon-Dong), Nam-gu, Busan 48434, Republic of Korea; suresh@ks.ac.kr; 4 The Center of Excellence «VERITAS», D. Serikbayev East Kazakhstan Technical University, D. Serikbayev St. 19, Ust-Kamenogorsk 070004, Kazakhstan; msadenova@edu.ektu.kz; 5 School of Digital Technologies and Artificial Intelligence, D. Serikbayev East Kazakhstan Technical University, D. Serikbayev St. 19, Ust-Kamenogorsk 070004, Kazakhstan

**Keywords:** scaffold, bone tissue engineering, additive manufacturing, mechanical properties, finite element analysis, computational fluid dynamics

## Abstract

**Highlights:**

- Porous scaffold mechanical properties match host bones to mitigate stress shielding.
- Post-processing of Ti6Al4V porous scaffolds via oxalic acid chemical etching.
- A finite element model predicts the modulus of elasticity with an accuracy of 8.4%.
- Computational fluid dynamics confirm adequate nutrient transport in scaffolds.
- A rational scaffold design can be tailored to materials and clinical applications.

**Abstract:**

This study investigates the fabrication, characterization, and computational analysis of a Ti6Al4V porous scaffold for bone tissue engineering (BTE). The main objective is to address the stress-shielding effect caused by the mismatch in the mechanical properties between the scaffold and surrounding bone. An octet-truss architecture was considered to design a highly porous scaffold (with 80.5% porosity) and fabricated using selective laser melting (SLM). The scaffold was then treated with post-processing chemical etching in oxalic acid to remove surface defects and tailor topography. Scanning electron microscopy (SEM) and X-ray diffraction (XRD) revealed that etching effectively removed adhered unmelted powder particles and created a distinct micro-textured strut surface (with increased roughness) that is conducive to osseointegration. The etching process also uniformly thinned down the struts and resulted in 10% mass loss. A compression test gave the scaffold’s compliance-corrected elastic moduli of 4.54 ± 0.18 GPa (pre-etching) and 3.53 ± 0.06 GPa (post-etching). These values closely match with the stiffness of human trabecular bone reported in the literature. The experimental modulus results were validated with a finite element model that predicted 4.188 GPa, which agrees well with the experiment. Furthermore, computational fluid dynamic simulations evaluated a permeability of 8 × 10^–9^ m^2^, consistent with transport in bone-like structures.

## 1. Introduction

Bone tissue engineering has emerged as an innovative research field to address the growing challenges associated with bone defects, fractures, and degenerative diseases amid a rapidly aging society. The main aim of this field is to develop artificial substitutes that can restore, maintain, or improve the biological and mechanical functions of damaged bone tissue. Synthetic biomaterials, particularly titanium alloys like Ti6Al4V, are gaining popularity in this field because of their good biocompatibility, high mechanical strength, corrosion resistance, and potential to induce osseointegration [1,2]. However, the modulus of elasticity of a Ti6Al4V (~110 GPa) implant is significantly higher than that of cortical bone (~10–30 GPa). This large difference causes poor stress distribution between the implant and the surrounding bone, which can lead to shielding effects and hinders the bone regenerative process [3].

The key part of BTE involves the development of three-dimensional (3D) porous structures, known as scaffolds, that mimic the natural bone. The fabrication of scaffolds with interconnected pore networks and customized mechanical properties is crucial for successful bone tissue engineering. Porous scaffolds with controlled architectures can match the stiffness of the human trabecular bone while providing the permeability for bone regeneration within the scaffold. Therefore, 3D scaffolds serve as templates for cell attachment, proliferation, and differentiation and ultimately support the formation of new bone tissue at defect sites [4,5]. However, the main challenge associated with the design and manufacture of scaffolds is creating them with mechanical properties that can be tailored to match those of the surrounding host bone, which is a critical factor in minimizing stress-shielding effects while maintaining structural integrity [6,7].

Recent advances in additive manufacturing (AM), such as electron beam melting (EBM) and selective laser melting techniques, provide an opportunity for the fabrication of Ti6Al4V scaffolds that are tailored for bone implants by enabling complex geometry [8]. AM techniques offer the following advantages compared to conventional manufacturing techniques: enabling excellent biocompatibility and osseointegration [9], allowing patient-specific customization and geometric complexity [10], and reducing the stress-shielding effect when the design parameters and process are carefully optimized [11]. Lu et al. [12] demonstrated that 3D-printed Ti6Al4V scaffolds produced by EBM achieved effective bone regeneration and expedited bone healing. Though EBM can produce complex geometric scaffolds, the surface roughness of implants produced from EBM are higher than for those generated from SLM, as SLM uses finer powder and a smaller laser spot size. This ensures SLM is highly suitable for producing implants of precise, intricate porous microstructures with high dimensional accuracy that are tuned for patient-specific customization.

In this study, SLM was selected because it offers unprecedented design freedom for fabricating complex porous architectures of different lattice structures with precise control over pore size, shape, and interconnectivity. We have chosen an octet-truss unit cell (having FCC lattice structure) configuration for porous scaffolds because of its high stiffness-to-weight ratio and its isotropic mechanical behavior [13]. However, the biological performance and corresponding osseointegration process of octet-truss scaffolds is significantly affected by surface morphology (such as residual powders and melt irregularities) [14].

The SLM process, while offering design flexibility, can cause various defects, such as porosity, residual stresses and the presence of unmelted powder particles on surfaces [15]. Excessive or uncontrolled roughness of the implant surface can create a favorable environment for bacterial colonization [16,17], which can establish biofilms on implant surfaces. This impedes the bone regeneration process and potentially delays osseointegration [18]. Furthermore, insufficient or suboptimal mechanical stability of the implant (caused by a mismatch of biomechanical properties between the scaffold and surrounding bone tissue) can lead to micro-mobility at the interface and, consequently, to bone resorption that prevents successful osseointegration [19].

To overcome these issues of SLM, post-processing treatments have gained significant attention recently. Mechanical post-processing or direct-access methods may be ineffective for the complex internal structures of porous scaffolds [20]. On the other hand, chemical polishing or etching methods can penetrate the internal volume of a porous structure, removing residual powder particles [21,22]. Chemical etching followed by SLM has been shown to result in improvements without significantly altering the underlying geometry [23,24]. It could remove defects such as adhered unmelted or partially melted powder particles from scaffolds’ surfaces [25], enhance mesenchymal stromal cell mineralization that accelerates osseointegration [26], and produce porous structures with different mechanical properties [21]. For the etching of titanium scaffolds, the following acid systems have been investigated: HF and HNO_3_ [21,27,28], H_2_SO_4_−HCL [29], HF [30], oxalic acid [31], and a mixture of oxalic acid and maleic acid [32,33].

The optimization of scaffold performance also requires comprehensive understanding of both mechanical strength and permeability behavior. While experimental testing has provided valuable insights into the macroscopic mechanical properties, finite element analysis (FEA) allows prediction of internal stress and strain distributions [34], which aids in scaffold optimization [35]. Similarly, computational fluid dynamics (CFDs) enable evaluation of scaffold permeability and wall shear-stress distributions, which are crucial for BTE applications, as high shear stresses prevent cell attachment and impede proliferation [36]. Experimental measurement of the permeability of such complex structures is often cumbersome, which makes CFDs a viable option.

Despite significant advances in AM, critical gaps remain in understanding the mechanical and permeability characteristics of Ti6Al4V octet-truss scaffolds. While many studies have focused on bending-dominated structures, this research investigates stretching-dominated octet-truss architecture. A significant gap in the current literature is the lack of an integrated approach that evaluates mechanical performance, fluid permeability, and surface morphology within a single study. This paper fills that gap by providing a holistic analysis of how SLM fabrication and subsequent chemical treatment affect these properties together. We introduce a refined experimental protocol using compliance correction to ensure accurate stiffness measurements, addressing common errors in testing high-porosity materials. Furthermore, this work utilizes a specialized thermo-chemical etching process with oxalic acid to sanitize internal lattice surfaces and create a beneficial micro-texture for bone growth. By validating computational models against experimental data, this study establishes a reliable framework for designing biomimetic scaffolds that balance mechanical support with the biological requirements for bone regeneration.

## 2. Materials and Methods

### 2.1. Scaffold Design and Fabrication

ELI-certified DIN EN ISO 22674 [37] Rematitan^®^ CL Ti6Al4V powder (Ispringen, Germany, Dentaurum GmbH & Co. KG.) was used for producing scaffold samples due to its proven biocompatibility and long history in the medical industry. Table 1 provides the chemical composition of the Rematitan^®^ CL powder. Rematitan^®^ CL powder has a spherical morphology with a particle size range of 10–53 µm.

The scaffolds were printed with an SLM machine, Concept Laser MLab Cusing R (Lichtenfels, Germany, Concept Laser GmbH.), equipped with an ytterbium fiber laser of a wavelength of 1070.3 nm and a standard deviation of 0.6 nm. The laser beam focus diameter was 50 µm and the maximum power was 100 W. An island scan strategy (divide a layer in the XY plane into squares of 5 mm × 5 mm) was applied to minimize the residual stress and improve uniformity. For each layer, the island pattern was shifted by 1 mm in the X and Y directions and rotated by 45°. The main 3D-printing parameters (including the overlap factors A1, A2, and A3) of the SLM machine are listed in Table 2. The hatch distance (A1 × W) was found to be 105 µm. Argon was used as a protective atmosphere, maintaining an oxygen level below 0.6% to prevent oxidation.

Volumetric energy density (ED), a commonly referred variable for process parameter optimization, is calculated by using the following relation:

(1) $$ED=\frac{P_{eff}}{v\times h\times d}\times 10^{6}J/{mm}^{3}$$

where P_eff_—laser power (W), v—scanning speed (mm/s), h—hatch distance (µm), and d—layer thickness (µm).

From the parameters of Table 2, the ED for scaffold production was around 45.24 J/mm^3^. This ED value ensures efficient melting of powder to produce Ti6Al4V samples with high density [38].

**Figure 1:**
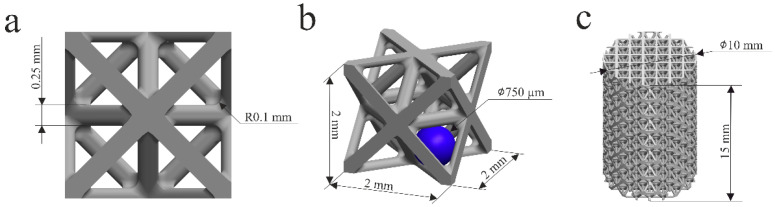
Octet-type porous scaffold parameters: (**a**) unit cell front view, (**b**) unit cell isometric view, and (**c**) mechanical testing scaffold sample.

In this work, an octet-truss unit cell (of a size of 2 mm × 2 mm × 2 mm) was designed in SolidWorks 2025 (Vélizy-Villacoublay, France, Dassault Systèmes SE). The beam thickness was 0.25 mm, and fillets of a radius of 0.1 mm were used at the beam intersection points to reduce stress concentration points; see Figure 1a. A scaffold model for mechanical testing was generated from the unit cell by Materialise Magics 23 (Leuven, Belgium, Materialise NV) software, and cylindrical samples (with diameters of ⌀10 mm and heights of 15 mm) were prepared, as shown in Figure 1c. To do this, a cylinder of the required size was created, and then the built-in software tools were used to fill its volume with the existing unit cell.

Porosity of the scaffold, P, is defined as the ratio of pore volume (empty space, V_p_) to the total unit cell volume, V (for a 2 mm m-sized unit cell, V = 8 mm^3^). P is defined by the following equation:

(2) $$P=\frac{V_{p}}{V}\times 100\%$$

Pore volume can be obtained from V_p_ = V − V_s_; where V_s_ = 1.56 mm^3^ is the solid volume of the octet octet-truss structure with a beam thickness of 0.25 mm and a fillet radius of 0.1 mm (measured directly from SolidWorks). Therefore, from Equation (2), the porosity of the scaffold was found to be 80.5%. The pore size of the structure was calculated as the diameter of a sphere inscribed within the void space between the beams [Figure 1b] and was 750 µm. The pore size falls within the optimal range for bone ingrowth and vascularization but could be tuned to meet specific clinical needs.

After 3D printing using an SLM machine, the samples were heat-treated in a vacuum oven. They were heated to 820 °C for 4 h, annealed for 1.5 h, and gradually cooled to 250 °C while maintaining same vacuum conditions.

### 2.2. Scaffolds Post-Processing

After heat treatment, all the samples were cut off from the printing bed and ultrasonically cleaned with distilled water. The samples were then etched to remove defects, primarily unmelted or adherent powder particles. Chemical etching was carried out in a 50 g/L oxalic acid solution using an ultrasonic bath at a temperature of 90 °C for 90 min. This process was selected because oxalic acid selectively dissolves loosely bound powder and surface irregularities while preserving the underlying microstructure. After etching, the samples were again cleaned ultrasonically. A significant amount of powder particles was precipitated after etching compared to the pre-etching ultrasonic cleaning.

### 2.3. Mechanical Testing

The compressive testing of the scaffolds was carried out in accordance with ISO 13314:2011 [39] (International Standard for Mechanical Testing of Metals, Ductility Testing, Compression Test for Porous and Cellular Metals) using the Shimadzu AG-X PLUS (Kyoto, Japan, Shimadzu Corporation) universal testing machine (10 kN load cell) at a cross speed of 1 mm/min. Three samples were tested both before and after etching, and the force and deformation were obtained at each position. The testing machine has its own inherent compliance (which means slight deformation of the testing system itself under load). This deformation is significant for highly porous materials such as scaffolds. Therefore, the compliance-correction method was applied for measuring the scaffold’s deformation more accurately. The machine’s compliance was determined by loading a massive steel block with a stiffness clearly exceeding the system’s stiffness. The resulting dependence of the machine’s deformation on load was subtracted from the experimental compression diagrams of the specimens. After obtaining the load-deformation data, the engineering stress–strain curves of the samples were plotted and the elastic modulus of each sample was evaluated from the linear region.

### 2.4. Characterization of the Scaffolds

The surface morphologies and microstructures of the scaffolds were examined using a TESCAN MIRA scanning electron microscope (Brno, Czech Republic, TESCAN). The dimensional measurements were made using a Mahr MarVision MM 420 microscope (Göttingen, Germany, Mahr GmbH). The phase-state identification of the structure was carried out using a PanAlytical X’Pert Pro diffractometer (Almelo, The Netherlands, PANalytical B.V.) that was equipped with a 100 µm detector hole and the anode material Cu/K-Alpha of a wavelength of 1.5406 Å. Bulk samples of a size of 10 × 10 × 3 mm were used with the same manufacturing and heat-treatment conditions. The samples were etched with Kroll’s reagent (2 mL HF, 4 mL HNO_3_, and 94 mL H_2_O) for 20 s to study the microstructure.

### 2.5. Finite Element Analysis of the Scaffolds

**Figure 2:**
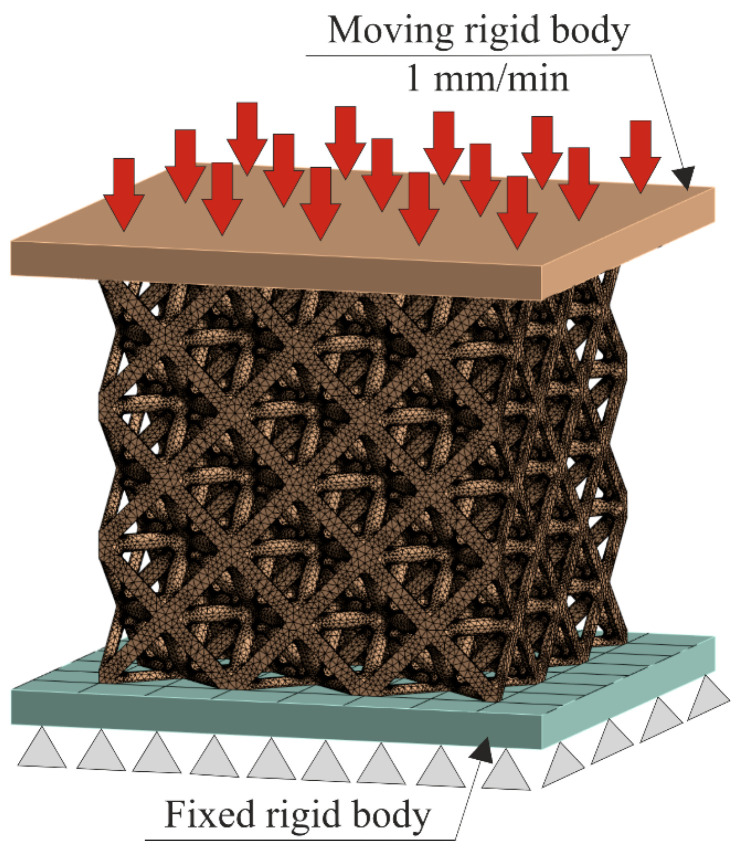
Boundary conditions of the porous scaffold’s finite element analysis.

FEA simulations were carried out by ANSYS 2024 R2 (Canonsburg, PA, USA, Ansys, Inc.) to predict the scaffold’s elastic modulus and stress distribution. Figure 2 shows the boundary conditions. The scaffold was placed between the upper and lower steel plates. The lower steel plate was fully fixed, while the upper plate was uniformly loaded normally at a rate of 1 mm/min, which corresponds to the loading-rate compression-testing machine. A tetrahedral mesh was used for the scaffold, while quadrilateral elements were considered for the upper and lower plates. The total numbers of elements and nodes were 3,181,297 and 5,001,976, respectively. A bilinear isotropic hardening model was used to describe the scaffold material (Ti6Al4V) behavior. Table 3 provides the details of the material properties used in the simulation process. Because of the low loading rate, the whole simulation process can be regarded as a quasi-static compression.

### 2.6. Computational Fluid Dynamics

CFD simulations were performed using ANSYS 2024 R2 CFX (Canonsburg, PA, USA, Ansys, Inc.) software for predicating permeability, wall shear stress (WSS), and velocity distribution inside the scaffold. To minimize the computational time, only one unit cell was selected, and symmetry boundary conditions, due to the repeatability (periodic nature) of the scaffold structure, were applied. The following Navier–Stokes equation [36] was used to solve the incompressible fluid (cell culture media with Dextran sulfate 5) flow through the scaffold:

(3) $$\rho \frac{\partial u}{\partial t}+\rho \left(u\times \nabla \right)u=-\nabla p+\mu \nabla^{2}u+\rho g$$

where

ρ—fluid density (ρ = 1050 kg/m^3^);

u—fluid velocity;

t—time;

μ—fluid viscosity (μ = 0.0037 kg/m/s);

g—external body force term such as gravity, electromagnetic forces, etc. (g = 0).

A normal uniform velocity of U = 0.1 mm/s was applied to the boundary at the inlet, a mass flow rate of $\dot{m}$ = 4.5 × 10^−7^ kg/s at the outlet, and no-slip boundary conditions at the scaffold’s walls. After conducting the mesh convergence study, an element size of 0.002 mm with 5 inflation layers was chosen at the walls to capture the WSS accurately. The mesh consisted of 4,741,067 elements and 1,504,112 nodes. Darcy’s law was used to predict the permeability, K=UμL/∆p, where $∆p=p_{in}-p_{out}$ is the difference between the inlet and outlet pressure.

## 3. Results and Discussion

### 3.1. Powder Characterization

**Figure 3:**
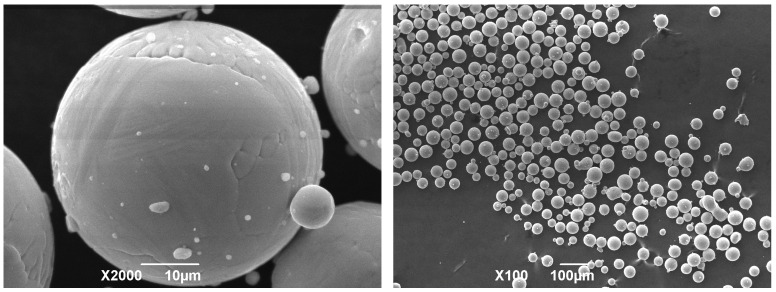
SEM images showing morphology of Rematitan^®^ CL (Ti6Al4V) powder used for SLM.

The morphology of the Rematitan^®^ CL powder used for the SLM is shown in Figure 3. Most of the powder particles are spherical in shape. The higher-magnification image reveals that smaller satellite particles are attached to the surfaces of larger ones, which are not completely smooth. It is also evident from the lower-magnification image that there is some agglomeration of the smaller particles. Overall, the lower-magnification image confirms the spherical nature of the powder, varied particle size distribution, and particle size claimed by the manufacturer. This spherical morphology (which is one of the characteristics of gas-atomized particles) enhances flowability and has high packing density, both of which are essential for consistent melt pool formation during SLM.

### 3.2. Scaffold Characterization

**Figure 4:**
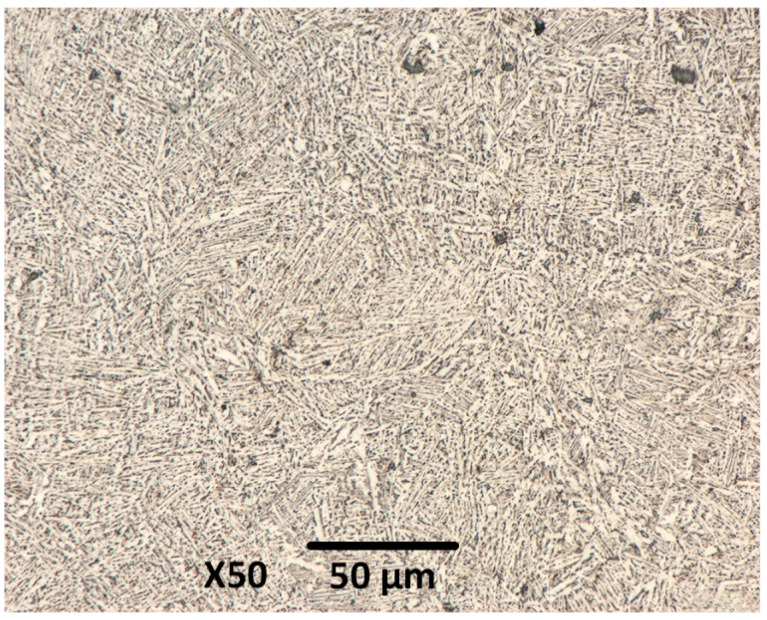
Microstructure of SLM-fabricated Ti6Al4V sample.

Figure 4 shows the microstructure of SLM-fabricated Ti6Al4V samples. The image reveals the formation of a fine lamellar α + β structure that resulted from the decomposition of α’ martensite during heat treatment after SLM. This transformation relieves the residual stresses generated from the SLM process and enhances the material’s ductility and fracture toughness. Consequently, the heat-treated samples achieved a more optimal balance between strength and toughness compared to the as-built state (exhibiting higher strength but more brittle in nature).

**Figure 5:**
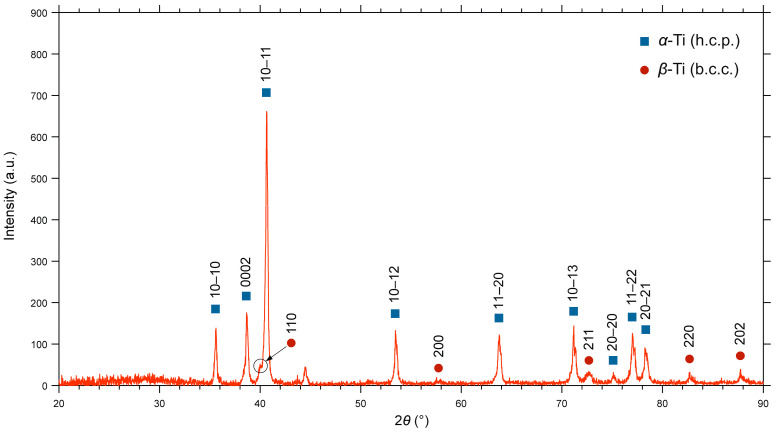
XRD of SLM-fabricated Ti6Al4V sample.

The X-ray diffraction [Figure 5] examination of the Ti6Al4V alloy provides critical understanding of its phase composition and crystallographic structure. XRD patterns revealed a dual-phase microstructure characteristic of this alloy system. The primary phase was identified as α-Ti, which exhibits a hexagonal close-packed (HCP) crystal structure, while the presence of β-Ti (secondary phase with BCC crystal structure) was also confirmed.

The relative intensities of the α- and β-phase peaks can vary significantly depending on the alloys’ thermomechanical history, including processing parameters and subsequent heat treatments. For example, rapid cooling from the β-phase field can result in the formation of a martensitic α’ phase, which appears as broadened HCP peaks in the XRD pattern. Precise analyses of peak positions and broadening can be used to estimate lattice strain and crystallite size that offers deeper insight into the material’s microstructural state.

**Figure 6:**
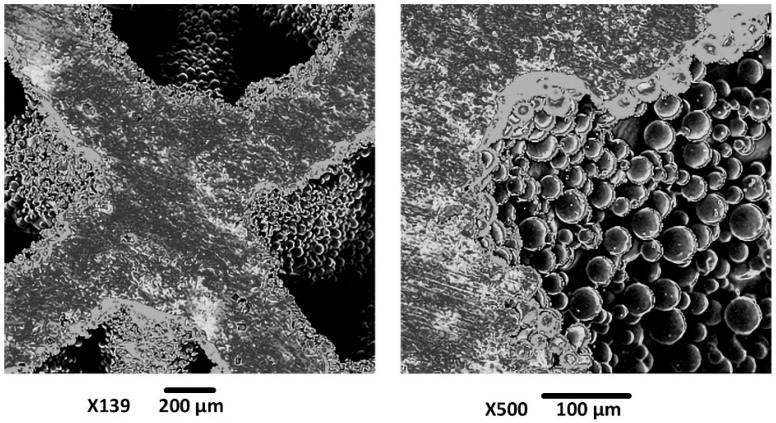
SEM image of the scaffold samples produced by SLM.

The SEM images of a scaffold sample produced by SLM are shown in Figure 6. The images reveal two minor manufacturing defects, although the overall structure of the scaffold remains intact. The observed defects are pronounced powder adhesion on the surface of the structure and the occurrence of a balling phenomenon.

The irregular surface topography of additive manufactured parts, characterized by partially melted powder particles, increases the residual powder adhesion. Strong interfacial forces between the powder and the substrate make it difficult to detach the adhered particles. These residue particles are a common artifact of the SLM process and are detrimental to the mechanical integrity and biocompatibility of the implant [40].

The balling phenomenon is an undesirable and commonly occurring defect in the SLM of metal powders. Balling refers to the agglomeration of molten powder into spherical droplets rather than a powder bed that is neither sintered nor completely melted, which typically occurs when the process and laser parameters are not chosen correctly [41].

**Figure 7:**
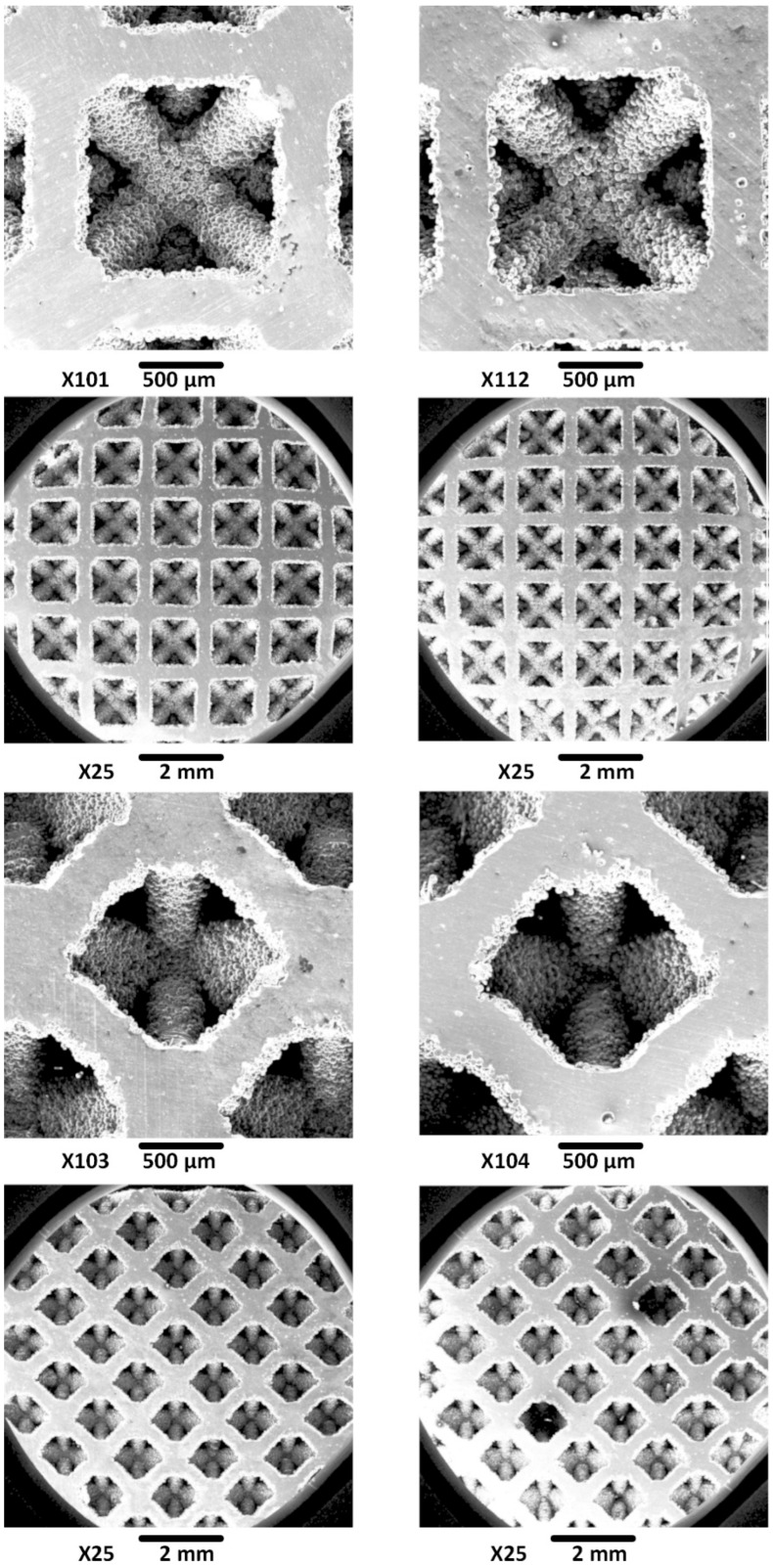
The SEM images of the scaffold samples before (**left**) and after (**right**) etching.

To mitigate the above-mentioned surface defects and enhance the surface topography, an etching process was employed. Figure 7 compares the Ti6Al4V scaffold before (left) and after (right) the chemical etching. Before etching, the struts exhibit a relatively smoother surface but are marred by partially melted and unmelted powder particles. These loosely adhered particles can lead to stress concentrations, premature fatigue failure, and potential release of debris into surrounding bone tissue. In addition to these powder residues, we can observe minor surface irregularities and porosity. The post-etching image shows a significant improvement in the surface quality, as most of the residue particles were removed. This resulted in a cleaner, more uniform surface, which is expected to enhance the fatigue life and mechanical performance of the scaffold.

**Figure 8:**
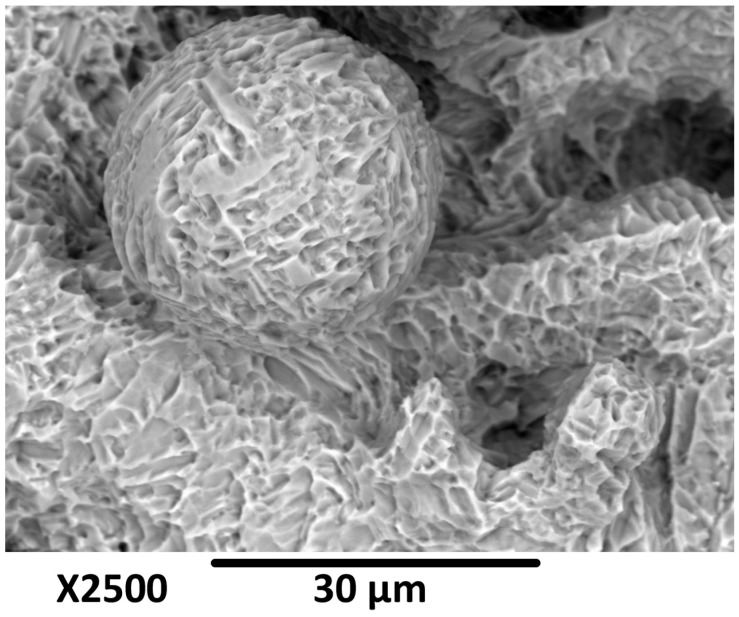
SEM image of Ti6Al4V powder particle after etching.

The etching process also induced a distinct micro-texture on the scaffold surface. The smooth surfaces of the struts before etching have been transformed into a more complex, textured topography (Figure 8). This increase in surface roughness at the microscopic level is beneficial for biomedical implants, as it promotes osteoblast (bone cell) attachment, proliferation, and differentiation. This new surface texture also increases the contact area and provides favorable environment for cell attachment, bone ingrowth, and, thereby, facilitates osseointegration. Therefore, the etching process serves a dual purpose: it not only removes the defects of the SLM process but also improves the surface topography so it is more conducive to biological bonding.

**Figure 9:**
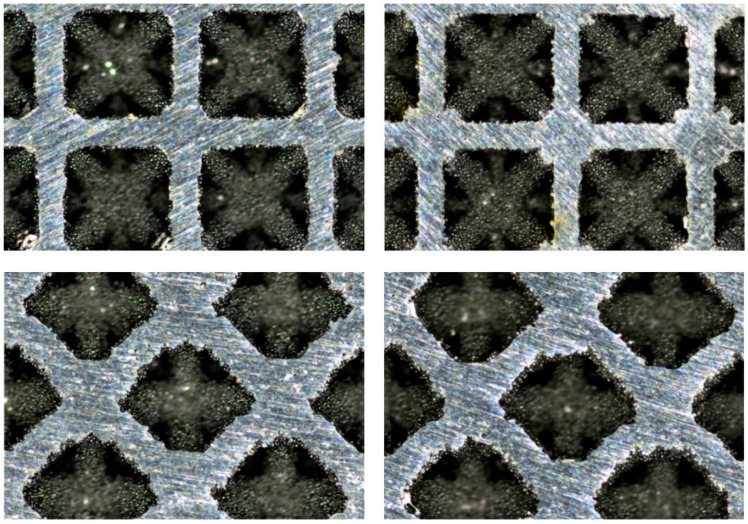
The measuring microscope images of the scaffold samples produced by SLM before (**left**) and after (**right**) etching.

The microscopic images in Figure 9 clearly visualize the effectiveness of the etching process. The dimensional features of the scaffolds pre- and post-etching are summarized in Table 4. The initial measurements of the as-built scaffolds revealed a noticeable anisotropy in strut thickness. The struts oriented vertically (45° to the build direction) had an average thickness of 364.8 ± 10.6 µm, whereas the horizontal ones were considerably thinner, with an average thickness of 289.5 ± 6.4 µm. This dimensional discrepancy is a common feature of the SLM process and is caused by several factors, such as layer-wise fabrication, melt pool dynamics, and heat distribution.

A consistent and significant reduction in the strut thickness was observed post-etching for both orientations. Table 4 also shows the percentage reduction in strut size and the corresponding mass loss. The thickness of the horizontal struts decreased by 9.9%, reaching an average of 260.8 ± 3.2 µm, while that of the vertical ones showed a reduction of 10.4%, with a final average thickness of 326.7 ± 6.9 µm. This uniform reduction in the strut thickness resulted in a total mass loss of the scaffold of 10.12%. These results indicate that the etching process removes the material isotopically at a relatively uniform rate irrespective of the orientation.

### 3.3. Mechanical Properties of the Scaffolds

**Figure 10:**
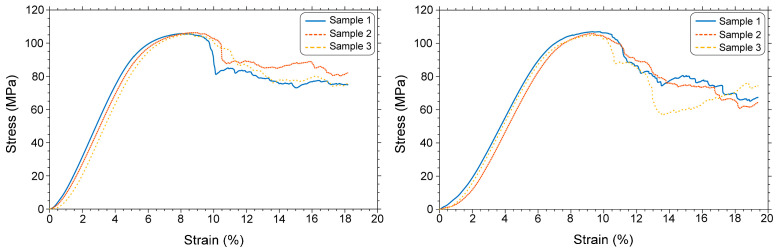
The compression test stress–strain graph of the octet-type porous scaffolds before (**left**) and after (**right**) etching.

Compression testing yielded the mechanical response of scaffold for suitable BTE applications. The elastic modulus of the scaffold could be obtained from the linear region of the stress–strain curve (Figure 10). Table 5 provides the values of the elastic moduli of three samples before and after etching without (raw data) and with the compliance correction method (that accounts for initial deformation). The raw elastic modulus was 2.165 GPa, whereas after applying the compliance correction method, the elastic modulus increased to 4.542 GPa. This value accurately reflects the true stiffness of the scaffold. Numerical simulation by using FEA predicted a modulus of 4.188 GPa (last column of Table 5), which is in good agreement with the experimental value. The difference between the experimental value and the FEA-predicted one is approximately 8.4%.

When compared to pre-etching (2.165 GPa), the elastic modulus of the post-etching (1.916 GPa) samples decreased by 11.5% without compliance correction and by 22.3% (4.542 GPa vs. 3.527 GPa) after compliance correction. This indicates that the etching slightly reduced the stiffness of the scaffold. If necessary, the FEM model can be modified to account for the reduction in stiffness during etching by changing the thickness of the rods.

The primary objective of bone scaffold design is to achieve mechanical properties compatible with the host bone tissue that promote effective regeneration. The elastic modulus of human bone varies widely depending on its type and anatomical location. Wu et al. [42] reported that trabecular bone has elastic moduli in the range of 1–22.3 GPa, while Choi et al. [43] demonstrated an average modulus of 4.59 GPa for human trabecular bone specimens. This value is consistent with the compliance-corrected elastic modulus of our scaffold, which was 4.542 GPa before etching. Our post-etching elastic modulus, 3.527 GPa, although significantly different from the pre-etching value, is within the range reported by Wu et al. [42]. This suggests that the stuffiness of the octet-strut scaffold developed in the present work is comparable to that of human trabecular or cancellous bone.

Achieving an appropriate modulus match is crucial. While dense cortical bone exhibits a higher elastic modulus, typically ranging from 10 to 30 GPa [44,45,46], a scaffold with such high stiffness can lead to stress shielding when implanted in less-dense bone regions. Stress shielding occurs when the implant bears an excessive portion of the physiological load, reducing mechanical stimulation to the surrounding bone and potentially leading to bone resorption and implant loosening [47]. By maintaining a lower elastic modulus relative to the cortical bone, the scaffold developed in the present work is highly suitable for trabecular bone regeneration (where the biomechanical compatibility of the scaffold encourages more physiological load transfer to the healing bone).

**Figure 11:**
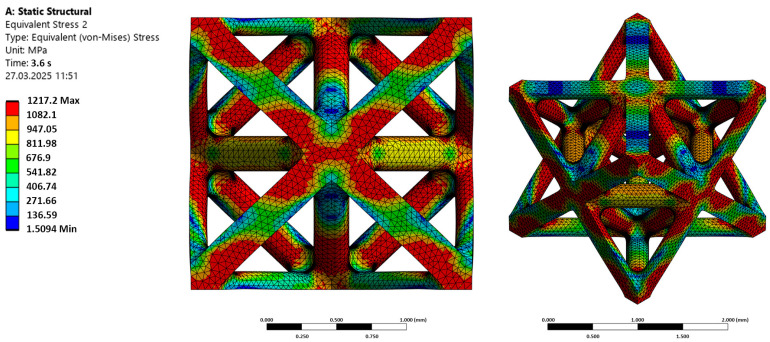
Distribution of von Mises equivalent stress in the octet-type porous scaffold. Shown in this figure are the front view (**left**) and isometric view (**right**) under static loading. Stress contours indicate relative stress levels, ranging from low (blue) to high (red).

It has been shown that pore architecture directs osteoconductivity, playing a critical role in the biological performance and bone tissue ingrowth of additively manufactured scaffolds [48]. FEA was conducted to investigate the mechanical response of the octet unit cell with filleted nodes for potential use as a bone scaffold in biomedical implants. Figure 11 shows the von Mises stress distribution obtained from static structural simulation for a one-unit cell. Both front (orthographic) and isometric views are provided to illustrate how the applied load is transmitted through the scaffold’s geometry.

The simulation results clearly reveal a non-uniform stress distribution throughout the octet unit cell. Regions of elevated stress (indicated by the red and orange contours) are concentrated at the nodal junctions where the individual struts intersect. These high-stress regions occur at both external corners and internal connection points, which indicates the nodal junctions act as physiological load transfer hubs. On the other hand, the mid-span regions of the struts, located away from the nodal junctions, exhibit considerably lower stress (represented by the blue and green contours).

While this structure provides useful porosity for tissue integration, the nature of the stress distribution highlights an important feature: the mechanical behavior under load is not uniform. Although the inclusion of fillets at the nodes reduces the effects of stress concentration, the stress concentration at the nodes corresponds to the mechanical behavior of lattice-type materials, and the node connections experience increased stress levels under load.

FEA analysis demonstrates the fundamental role of the nodes as primary load transfer points within the octet structure, which makes them inherently prone to localized stress buildup. The stress distribution visualization clearly identifies these high-stress zones as the critical regions susceptible to mechanical failure under operational loading. Understanding this pattern of stress distribution is essential for guiding further design refinements, such as optimizing the curvature of the nodes or adjusting strut thicknesses, to enhance the structural integrity and long-term endurance of the scaffold within the demanding biomechanical environment of the human body. Overall, this qualitative analysis provides valuable insight into the load transfer mechanisms and highlights the key areas of octet strut scaffolds’ optimum design process.

### 3.4. Permeability of the Scaffolds

**Figure 12:**
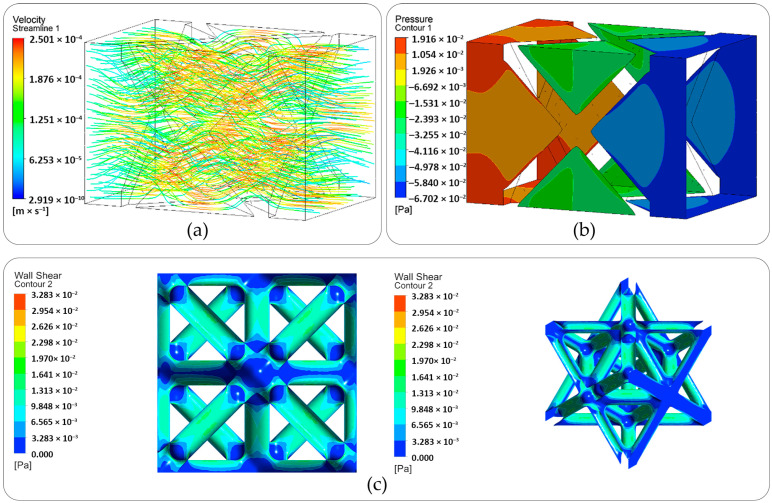
CFD simulation results for scaffold permeability: (**a**) fluid streamlines and velocity contours, (**b**) pressure drop across the structure and (**c**) wall shear stress in front and isometric views.

Figure 12 shows the CFD simulation results of streamlines and velocity Figure 12a and pressure distributions Figure 12b inside the scaffold Figure 12c wall shear stress at front and isometric views. As the flow is steady and laminar, we can observe that the pressure decreases uniformly from inlet to the outlet. The maximum velocity (around 0.25 mm/s) is higher than the inlet velocity, as the cross-sectional area inside the scaffold is reduced due to the complexity of the structure. The calculated wall shear stress values range from 0 to 3.28 × 10^−2^ Pa across the scaffold surface. The permeability of the scaffold was found to be around K = 8 × 10^−9^ m^2^, which is in good agreement with the literature for Ti6Al4V scaffolds [49,50]. The CFD analysis results combined with the experimental (mechanical testing) and FEA results confirm that the scaffold design satisfies both the mechanical and permeability behaviors required for bone tissue growth and regeneration.

## 4. Conclusions

This research successfully demonstrated that the selective laser melting fabrication and comprehensive characterization of Ti6Al4V scaffolds with an octet-truss architecture for bone tissue engineering applications effectively achieve the primary objective of obtaining the mechanical properties of the scaffold compatible with the host bone tissue to mitigate stress shielding.

In conclusion, this research demonstrates the following key results:

- The developed scaffolds possessed a high open porosity of 80.5%, providing an optimal environment for osteoconduction.
- The application of oxalic acid etching successfully removed partially melted powder particles and satellites from the internal surfaces of the scaffolds. Although this process resulted in a marginal reduction in strut thickness (approximately 10%), the overall mechanical integrity of the structures remained uncompromised. This surface modification is expected to enhance biological fixation and promote superior osseointegration.
- Following the etching process, the elastic modulus of the scaffolds was measured at 3.527 GPa. This value falls within the target biomechanical range of human trabecular bone, thereby minimizing the risk of the stress-shielding effect.
- The finite element analysis model predicted an elastic modulus within 8.4% of the experimental result, confirming the model’s accuracy and reliability. Furthermore, FEA simulations revealed a non-uniform stress distribution within the scaffold and identified stress concentrations at the nodal junctions, which makes them potential mechanical failure regions.
- Complementary computational fluid dynamics analysis predicted a permeability value of K = 8 × 10^−9^ m^2^, consistent with the literature data and indicative of adequate nutrient transport capability.

In summary, this study validates that combining SLM fabrication with controlled chemical etching enables the development of Ti6Al4V scaffolds with elastic moduli compatible with the human trabecular bone, improved surface biocompatibility, and reliable mechanical performance. This integrated experimental and computational approach provides a robust framework for designing scaffolds with required stiffness and improved biological integration for orthopedic implants.

However, comprehensive in vitro and in vivo testing is necessary for a comprehensive assessment of the biological potential of the developed scaffolds. Cell culture studies will allow the determination of the adhesion and viability parameters of osteoblasts directly on the surface of the formed structures. Further studies in animal models are required to definitively confirm the osseointegration properties and the material’s ability to form stable biomechanical contact with living bone tissue.

## Figures and Tables

**Table 1:** Chemical composition of metal powder.

Component	Content (%)
Ti	90
Al	6
V	4
Other components < 1%: N, C, H, Fe, O.	

**Table 2:** Parameters for selective laser melting process.

Parameter	Abbreviation	Setting
Power	P (W)	95
Scanning speed	v (mm/s)	800
Layer thickness	d (µm)	25
Track width	W (µm)	150
Track overlap factor A1	A1	0.7
Island overlap factor A2	A2	0.15
Island overlap factor A3	A3	0.15
Hatch distance	h (µm)	105

**Table 3:** Material properties used in the simulation process.

Material	Density (kg/m^3^)	Young’s Modulus (GPa)	Yield Strength (MPa)	Poisson’s Ration	Tangent Modulus (GPa)
Ti6Al4V	4405	107	1098	0.323	1.332

**Table 4:** Measured strut size of SLM-produced octet-type scaffolds.

Direction	Measured Strut Size			Mass Loss
	Before Etching	After Etching	Reduction	
Horizontal cross-section	289.5 ± 6.4	260.8 ± 3.2	9.9%	10.12%

**Table 5:** Mechanical testing and finite element analysis results.

Sample	Young’s Modulus Test (GPa)	Young’s Modulus Test with Compliance Correction (GPa)	Young’s Modulus Test After Etching (GPa)	Young’s Modulus Test After Etching with Compliance Correction (GPa)	Young’s Modulus, FEA-Predicted (GPa)
Sample 1	2.196	4.713	1.899	3.458	
Sample 2	2.120	4.361	1.931	3.562	4.188
Sample 3	2.180	4.551	1.917	3.563	
Average	2.165 ± 0.040	4.542 ± 0.176	1.916 ± 0.016	3.527 ± 0.060	4.188

## Data Availability

The original contributions presented in this study are included in the article, and further inquiries can be directed to the corresponding author.

## Abbreviations

The following abbreviations are used in this manuscript:

BTE	Bone tissue engineering
SLM	Selective laser melting
SEM	Scanning electron microscopy
XRD	X-ray diffraction
3D	Three-dimensional
AM	Additive manufacturing
EBM	Electron beam melting
FEA	Finite element analysis
CFDs	Computational fluid dynamics
ED	Energy density
WSS	Wall shear stress
HCP	Hexagonal close-packed
FCC	Face-centered cubic
BCC	Body-centered cubic
